# In Vitro Toxicity of Chinese Russell’s Viper (*Daboia siamensis*) Venom and Neutralisation by Antivenoms

**DOI:** 10.3390/toxins14070505

**Published:** 2022-07-20

**Authors:** Mimi Lay, Qing Liang, Geoffrey K. Isbister, Wayne C. Hodgson

**Affiliations:** 1 Monash Venom Group, Department of Pharmacology, Biomedical Discovery Institute, Monash University, Clayton, VIC 3800, Australia; mimi.lay@monash.edu (M.L.); qing.liang@monash.edu (Q.L.); geoff.isbister@gmail.com (G.K.I.); 2 Department of Emergency Medicine, The First Affiliated Hospital of Guangzhou Medical University, 151 Yanjiang Rd., Guangzhou 510120, China; 3 Clinical Toxicology Research Group, University of Newcastle, Callaghan, NSW 2308, Australia

**Keywords:** snake, venom, antivenom, Russell’s viper, neurotoxicity, myotoxicity, *Daboia siamensis*

## Abstract

*Daboia siamensis* (Russell’s viper) is a highly venomous and medically important snake in China, as well as much of Asia. There is minimal information on the pharmacological activity of the venom of the Chinese species, and currently no commercially available specific antivenom in China. This has led to the use of non-specific antivenoms to treat *D. siamensis* envenomation. In this study, the in vitro neurotoxicity and myotoxicity of *D. siamensis* venom was examined and the efficacy of four antivenoms was investigated, including the recently developed Chinese *D. siamensis* monovalent antivenom (C-DsMAV) and three commercially available antivenoms (Thai *D. siamensis* (Thai-DsMAV) monovalent antivenom, *Deinagkistrodon acutus* monovalent antivenom (DaAV), and *Gloydius brevicaudus* monovalent antivenom (GbAV). *D. siamensis* venom (10–30 µg/mL) caused the concentration-dependent inhibition of indirect twitches in the chick biventer cervicis nerve muscle preparation, without abolishing contractile responses to exogenous agonists ACh or CCh, indicating pre-synaptic neurotoxicity. Myotoxicity was also evident at these concentrations with inhibition of direct twitches, an increase in baseline tension, and the partial inhibition of ACh, CCh, and KCl responses. The prior addition of C-DsMAV or Thai-DsMAV prevented the neurotoxic and myotoxic activity of *D. siamensis* venom (10 µg/mL). The addition of non-specific antivenoms (GbAV and DaAV) partially prevented the neurotoxic activity of venom (10 µg/mL) but failed to neutralize the myotoxic effects. We have shown that *D. siamensis* venom exhibits in vitro weak presynaptic neurotoxicity and myotoxicity, which can be prevented by the pre-addition of the Chinese and Thai Russell’s viper antivenoms. Non-specific antivenoms were poorly efficacious. There should be further development of a monospecific antivenom against *D. siamensis* envenomation in China.

## 1. Introduction

The Russell’s viper is a large venomous snake with widespread geographical distribution across many Asian countries. A number of subspecies of Russell’s viper have been described, i.e., *Daboia russelii russelii* (India, Pakistan, and Bangladesh), *D. r. pulchella* (Sri Lanka and South India), *D. r. siamensis* (Cambodia, Myanmar, Thailand, Southern China), *D. r. formosensis* (Taiwan), and *D. r. limitis* and *sublimitis* (Indonesia and Java, Indonesia, respectively). However, these species are now widely recognised as two species i.e., *D. russelii* and *D. siamensis*, with the latter nomenclature used for all Russell’s vipers to the east of the Bay of Bengal [1,2,3,4].

Haemostatic disturbances (i.e., venom-induced consumptive coagulopathy, haemorrhage, and thrombotic microangiopathy) and acute renal failure are important clinical effects of *D. siamensis* envenomation, irrespective of the geographical location of the biting species. However, other clinical outcomes, including local swelling, blistering ecchymosis, hypopituitarism, generalised capillary permeability, neurotoxicity, and myotoxicity, are geographically disjunct [5,6,7,8,9,10,11]. Comparative proteomic studies elucidated the diverse composition of *Daboia* species venoms from different regions of South-East Asia, supporting the variable clinical effects observed following snake envenomation from different locations [10,12,13,14,15,16,17,18,19,20].

Previous studies have almost exclusively focused on the coagulopathic and nephrotoxic effects of the venom which, as indicated previously, are the key problems following *D. siamensis* envenomation [21,22,23]. However, the literature pertaining to the neurotoxic and myotoxic effects of various *D. siamensis* venoms is considerably less developed. It has been proposed that neuromuscular paralysis and mild skeletal muscle damage are unique to the Sri Lankan species [9,10,11]. Populations of *D. siamensis* in Myanmar and Thailand have not been known to cause significant, if any, neuromuscular paralysis, or systemic myotoxicity [21,24]. The literature on Taiwanese species is less consistent, with infrequent clinical reports of neuro-myotoxic envenomation [6,19,25].

Biochemical and pharmacological studies have shown that venoms from almost all Russell’s vipers, despite their geographical origin, have a large quantity of phospholipase A_2_ toxins (PLA_2_) [10,13,15,16,17,18,19,20,26]. Previously, our laboratory has demonstrated that pre-synaptic neurotoxicity and myotoxicity caused by Sri Lankan *D. russelii* venom are largely due to the actions of U1-viperitoxin Dr-1a and U1-viperitoxin-Dr1b [9,10]. These toxins are thought to be responsible for the clinical neurotoxicity and mild myotoxicity in envenomed patients [9,10,11], as opposed to *D. siamensis* populations, which exhibit equivocal neurotoxic and myotoxic manifestations in humans.

The most effective treatment for systemic snake envenomation is the administration of antivenom. The absence of specific antivenoms that are raised against the venom of the biting snake for many species means that non-specific antivenoms (i.e., raised against the venom(s) from different genus/species) are often used. Even the effectiveness of specific antivenoms may be reduced by inter- and intra-species variation in venom composition. Despite this, many studies have demonstrated that antivenoms can neutralise the venoms from closely related species, or the same species found across different geographical locations [13,21,27,28,29].

*D. siamensis* is a medically important species in China, yet commercial antivenoms are only available against species in India, Myanmar, Taiwan, and Thailand. Pre-clinical studies and immunobinding assays suggest that *D. siamensis* monovalent antivenom from Thailand prevents in vitro coagulopathy and lethality in mice caused by the Chinese species, albeit the efficacy of the antivenom was lower than against the effects of venom sourced from the same location [21]. Another study also showed some cross-reactivity between Taiwanese *D. siamensis* monovalent antivenom and Guangxi *D. siamensis* venom due to shared conserved antigenicity of major toxins [16].

As a specific antivenom against *D. siamensis* is not available in Southern China, antivenoms raised against the venoms of other viperid species, i.e., the Chinese sharp-nosed pit viper (*Deinagkistrodon acutus*) and short-tailed pit viper (*Gloydius brevicaudus*), have been used, either alone or in combination. Although these antivenoms are currently recommended for the treatment of the coagulopathic effects in patients envenomed by *D. siamensis*, there is considerable debate regarding the clinical effectiveness against other venom toxicities. Recently, the Shanghai Serum Biotechnology company has produced a monovalent antivenom that has been raised against the venom of Chinese *D. siamensis*, although not yet available for human use.

The effects of Chinese *D. siamensis* venom have been poorly studied in comparison to Russell’s viper venoms from other regions. Given that neurotoxicity and myotoxicity are potentially important clinical effects of envenomation by some geographical variations of *Daboia* species, the aim of this study was to determine the effects of Chinese *D. siamensis* venom in an in vitro skeletal muscle preparation. We also assessed the in vitro efficacy of the recently developed Chinese monovalent antivenom in comparison to the commercially available Thai Queen Saovabha Memorial Institute (QSMI) *D. siamensis* monovalent antivenom. In addition, we investigated the protective effects of two additional non-specific pit viper antivenoms.

## 2. Results

### 2.1. In Vitro Neurotoxicity

**Figure 1:**
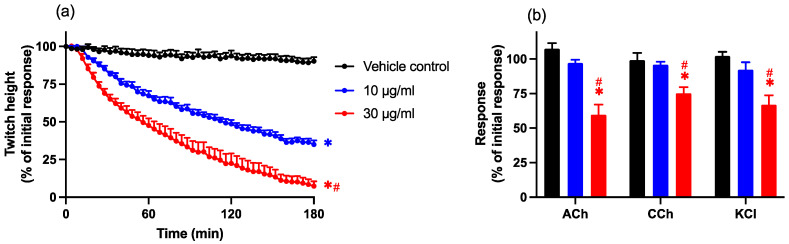
The concentration-dependent neurotoxic effects of *D. siamensis* venom on (**a**) indirect twitches and (**b**) contractile responses to exogenous agonists ACh (1 mM), CCh (20 µM), and KCl (40 mM) in the chick biventer cervicis nerve–muscle preparation. Data presented as mean ± SEM, * *p* < 0.05, significantly different from the vehicle control; # *p* < 0.05, significantly different from venom (10 µg/mL), one-way ANOVA followed by Bonferroni’s post-hoc test. *N* = 6, where *n* is the number of preparations from different animals.

Chinese *D. siamensis* venom (10 and 30 µg/mL) caused the concentration-dependent inhibition of indirect twitches of the chick biventer preparation over a 3 h period (*n* = 6; one-way ANOVA, *p* < 0.05; Figure 1a). Venom potency was determined by calculating the t_50_ (i.e., the time taken to reach 50% twitch inhibition) which was 114 ± 9 min for 10 µg/mL and 60 ± 13 min for 30 µg/mL. Venom, at the lower concentration (10 µg/mL), did not significantly inhibit contractile responses to exogenous agonists ACh, CCh, and KCl (Figure 1b), indicative of pre-synaptic neurotoxic activity. At the higher concentration (30 µg/mL), there was a small but significant decrease in the post-venom response to all agonists, indicative of myotoxic activity.

### 2.2. In Vitro Myotoxicity

**Figure 2:**
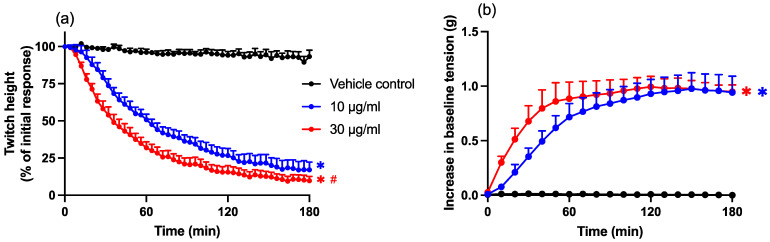
The concentration-dependent myotoxic effects of *D. siamensis* venom on (**a**) direct twitches and (**b**) baseline tension in the chick biventer cervicis nerve–muscle preparation. Data presented as mean ± SEM, * *p* < 0.05, significantly different from the vehicle control; # *p* < 0.05, significantly different from venom (10 µg/mL), one-way ANOVA followed by Bonferroni’s post-hoc test. *N* = 6, where *n* is the number of preparations from different animals.

Chinese *D. siamensis* (10 and 30 µg/mL) caused the concentration-dependent inhibition of direct twitches of the chick biventer preparation, with an associated increase in baseline tension over a 3 h period, indicative of myotoxicity (*n* = 6; one-way ANOVA, *p* < 0.05; Figure 2a,b). Venom potency was determined by calculating the t_50_ (i.e., time taken to reach 50% twitch inhibition) with values of 59 ± 6 min for 10 µg/mL and 36 ± 5 min for 30 µg/mL.

### 2.3. In Vitro Antivenom Protection Studies

#### 2.3.1. Unfiltered Versus Filtered Antivenoms

In preliminary testing, Chinese *D. siamensis* monovalent antivenom and *G. brevicaudus* antivenoms had a small but significant inhibitory effect on the twitch height (*n* = 3–4, data not shown). Therefore, all antivenoms were filtered (see Methods) prior to use to remove contaminants that may be having an effect on the tissues. The neutralising capacity of the antivenoms was not reduced by this process.

#### 2.3.2. Chinese *D. siamensis* Monovalent Antivenom: In Vitro Neurotoxicity Antivenom Protection

**Figure 3:**
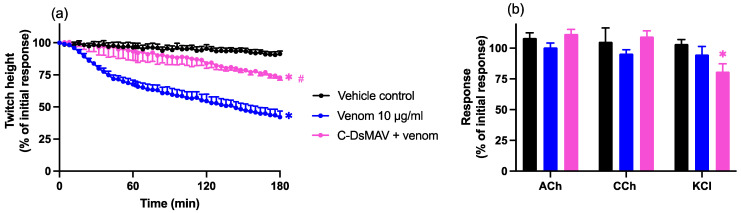
Effects of Chinese *D. siamensis* monovalent antivenom (C-DsMAV) on *D. siamensis* venom (10 µg/mL) induced neurotoxicity in the chick biventer cervicis nerve–muscle preparation. Effects of venom, in the presence and absence of antivenom, on (**a**) indirect twitches and (**b**) contractile responses to exogenous agonists; ACh (1 mM), CCh (20 µM) and KCl (40 mM). (**a**) * *p* < 0.05, significantly different to the vehicle control; # *p* < 0.05, significantly different to venom in the absence of antivenom, one-way ANOVA followed by Bonferroni’s post-hoc test. (**b**) * *p* < 0.05, significantly different to the pre-venom response, paired *t*-test; *N* = 5–6, where *n* is the number of preparations from different animals.

In the absence of antivenom, Chinese *D. siamensis* venom (10 µg/mL) significantly inhibited indirect twitches without affecting contractile responses to exogenous agonists. The prior addition of Chinese *D. siamensis* monovalent antivenom (15 µL; 3× the recommended concentration) markedly attenuated the inhibition of indirect twitches (*n* = 5–6; one-way ANOVA; *p* < 0.05; Figure 3a), although the twitches were still reduced compared to the vehicle control. Venom alone did not significantly inhibit the agonist response, and although the pre-addition of antivenom prevented a slight decrease in the ACh and CCh response, this effect was not statistically significant compared to venom alone (*n* = 5–6; one-way ANOVA; *p* < 0.05; Figure 3b). Antivenom alone did not have any significant effect on indirect twitches (data not shown), but the combination with venom caused a slight decrease in the KCl response when compared to vehicle, but not when compared to venom alone (*n* = 5–6; paired *t*-test; Figure 3b).

#### 2.3.3. Chinese *D. siamensis* Monovalent Antivenom: In Vitro Myotoxicity Antivenom Protection

**Figure 4:**
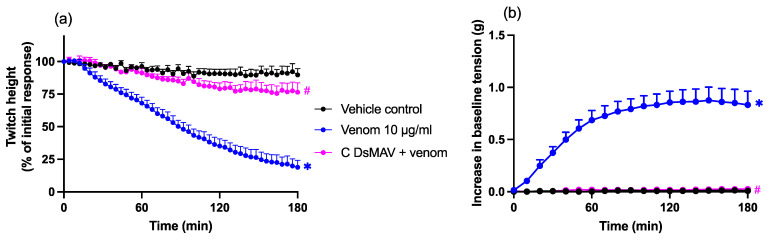
Effects of Chinese *D. siamensis* monovalent antivenom (C-DsMAV) on *D. siamensis* venom (10 µg/mL) induced myotoxicity in the chick biventer cervicis nerve–muscle preparation. Effects of venom, in the presence and absence of antivenom, on (**a**) direct twitches and (**b**) baseline tension. * *p* < 0.05, significantly different to the vehicle control; # *p* < 0.05, significantly different to venom in the absence of antivenom, one-way ANOVA followed by Bonferroni’s post-hoc test. *N* = 5–6, where *n* is the number of preparations from different animals.

Chinese *D. siamensis* venom (10 µg/mL) inhibited direct twitches with an associated increase in baseline tension, indicative of myotoxicity. The prior addition of Chinese *D. siamensis* monovalent antivenom (20 µL; 4× the recommended concentration) prevented a reduction in twitches and an increase in baseline tension (*n* = 5–6; one-way ANOVA; *p* < 0.05; Figure 4a,b).

#### 2.3.4. Thai *D. siamensis* Monovalent Antivenom: In Vitro Neurotoxicity Antivenom Protection

**Figure 5:**
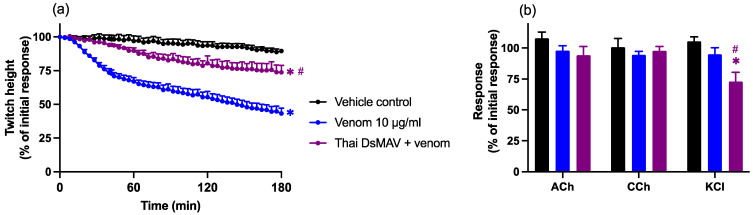
Effects of Thai *D. siamensis* monovalent antivenom (Thai DsMAV) on *D. siamensis* venom (10 µg/mL) induced neurotoxicity in the chick biventer cervicis nerve–muscle preparation. Effects of venom, in the presence and absence of antivenom, on (**a**) indirect twitches and (**b**) contractile responses to exogenous agonists; ACh (1 mM), CCh (20 µM), and KCl (40 mM). (**a**) * *p* < 0.05, significantly different to the vehicle control; # *p* < 0.05, significantly different to venom in the absence of antivenom, one-way ANOVA followed by Bonferroni’s post-hoc test. (**b**) * *p* < 0.05, significantly different to the pre-venom response, paired *t*-test; # *p* < 0.05, significantly different to venom in the absence of antivenom, one-way ANOVA, followed by Bonferroni’s post-hoc test. *N* = 5, where *n* is the number of preparations from different animals.

The prior addition of Thai *D. siamensis* monovalent antivenom (85 µL; 2× the recommended concentration) significantly prevented a reduction in indirect twitches caused by Chinese *D. siamensis* venom (10 µg/mL) (*n* = 5; one-way ANOVA; *p* < 0.05; Figure 5a), indicating a partial protective effect of the venom at this concentration. Antivenom alone did not have any effects on indirect twitches (data not shown), but did cause a small but significant reduction in the KCl response when compared to venom alone (one-way ANOVA; *p* < 0.05; Figure 5b).

#### 2.3.5. Thai *D. siamensis* Monovalent Antivenom: In Vitro Myotoxicity Antivenom Protection

**Figure 6:**
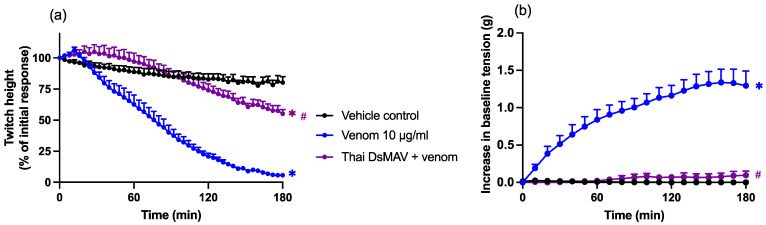
Effects of Thai *D. siamensis* monovalent antivenom (Thai DsMAV) on *D. siamensis* venom (10 µg/mL) induced myotoxicity in the chick biventer cervicis nerve–muscle preparation. Effects of venom, in the presence and absence of antivenom, on (**a**) direct twitches and (**b**) baseline tension. * *p* < 0.05, significantly different to the vehicle control; # *p* < 0.05, significantly different to the venom in the absence of antivenom, one-way ANOVA, followed by Bonferroni’s post-hoc test. *N* = 5–6, where *n* is the number of preparations from different animals.

The prior addition of Thai *D. siamensis* monovalent antivenom (85 µL; 2× the recommended concentration) markedly attenuated the inhibition of direct twitches caused by Chinese *D. siamensis* venom (10 µg/mL), although the twitches were still reduced compared to the vehicle control (*n* = 5–6; one-way ANOVA; *p* < 0.05; Figure 6a). In addition, Thai antivenom prevented an increase in baseline tension caused by the venom (*n* = 5; one-way ANOVA; *p* < 0.05; Figure 6b).

#### 2.3.6. Chinese Antivenom: In Vitro Neurotoxicity Antivenom Protection

**Figure 7:**
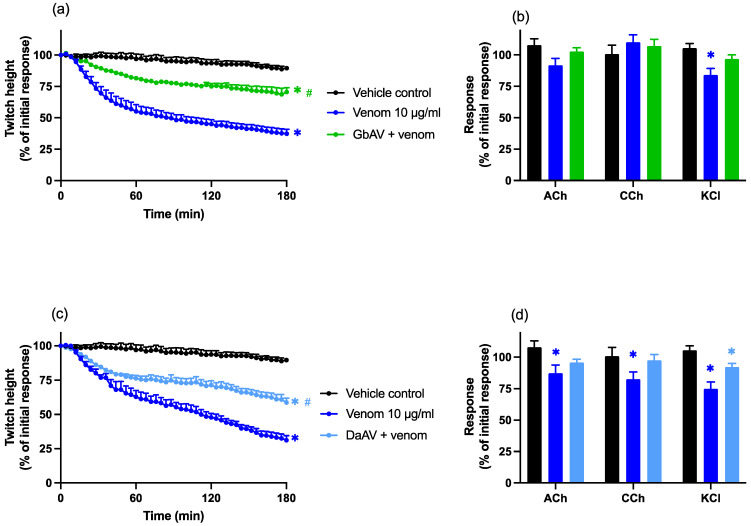
Effects of (**a**,**b**) Chinese *G. brevicaudus* monovalent antivenom (GbAV; 150 µL; 2× the recommended concentration) or (**c**,**d**) Chinese *D. acutus* antivenom (DaAV; 35 µL; 3× the recommended concentration) in the chick biventer cervicis nerve–muscle preparation. Effects of venom, in the presence and absence of Chinese GbAV, on (**a**) indirect twitches and (**b**) contractile responses to exogenous agonists; ACh (1 mM), CCh (20 µM), and KCl (40 mM). Effects of venom, in the presence and absence of Chinese DaAV, on (**c**) indirect twitches and (**d**) contractile responses to exogenous agonists; ACh (1 mM), CCh (20 µM), and KCl (40 mM). (**a**,**c**) * *p* < 0.05, significantly different to the vehicle control; # *p* < 0.05, significantly different to venom in the absence of antivenom, one-way ANOVA followed by Bonferroni’s post-hoc test. (**b**,**d**) * *p* < 0.05, significantly different to pre-venom response, paired *t*-test; # *p* < 0.05, significantly different to venom in the absence of antivenom, one-way ANOVA followed by Bonferroni’s post-hoc test. *N* = 5–6, where *n* is the number of preparations from different animals.

The prior addition of Chinese *G. brevicaudus* antivenom (150 µL; 2× the recommended concentration; GbAV), or Chinese *D. acutus* antivenom (35 µL; 3× the recommended antivenom concentration; DaAV) partially prevented the neurotoxic effects of venom, compared to venom alone (*n* = 5–6; one-way ANOVA; *p* < 0.05; Figure 7a,c). However, the twitches were still significantly reduced compared to the control (*n* = 5–6; one-way ANOVA; *p* < 0.05; Figure 7b,d). Although there was restoration of KCl responses in the presence of *G. brevicaudus* antivenom, this effect was not significant (*n* = 5–6; one-way ANOVA; *p* < 0.05; Figure 7b).

#### 2.3.7. Chinese Antivenom: In Vitro Myotoxicity Antivenom Protection

**Figure 8:**
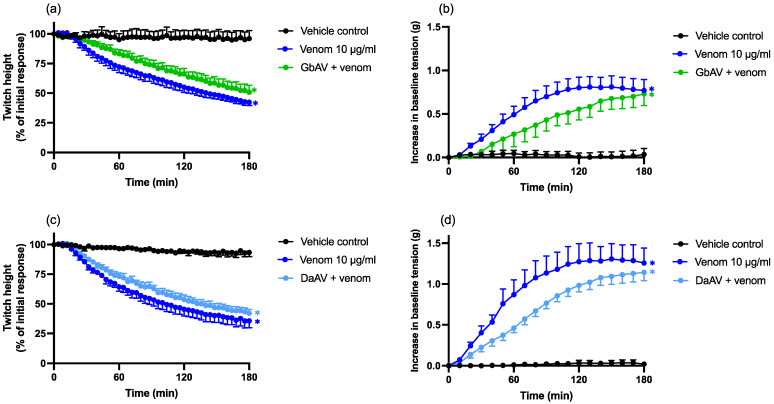
Effects of (**a**,**b**) Chinese *G. brevicaudus* monovalent antivenom (GbAV; 150 µL; 2× the recommended concentration) or (**c**,**d**) Chinese *D. acutus* antivenom (DaAV; 35 µL; 3× the recommended concentration) in the chick biventer cervicis nerve–muscle preparation. Effects of venom, in the presence and absence of Chinese GbAV, on (**a**) direct twitches and (**b**) baseline tension. Effects of venom, in the presence and absence of Chinese DaAV, on (**c**) direct twitches and (**d**) baseline tension. * *p* < 0.05, significantly different to the vehicle control; one-way ANOVA followed by Bonferroni’s post-hoc test. *N* = 4–5, where *n* is the number of preparations from different animals.

The prior addition of Chinese *G. brevicaudus* antivenom (150 µL; 2× the recommended concentration; GbAV) or Chinese *D. acutus* antivenom (35 µL; 3× the recommended antivenom concentration; DaAV) failed to prevent the myotoxic effects of venom, compared to venom alone (*n* = 4–5; one-way ANOVA; *p* < 0.05; Figure 8a,c). In addition, neither Chinese *G. brevicaudus* antivenom (150 µL; 2× the recommended concentration; GbAV) nor Chinese *D. acutus* antivenom (35 µL; 3× the recommended antivenom concentration; DaAV) antivenom prevented an increase in baseline tension caused by the venom (*n* = 4–5; one-way ANOVA; *p* < 0.05; Figure 8b,d).

## 3. Discussion

We examined the in vitro neurotoxic and myotoxic activity of Chinese *D. siamensis* venom and the efficacy of different specific and non-specific antivenoms. We demonstrate the neutralisation efficacy of Chinese *D. siamensis* monovalent antivenom against *D. siamensis* venom. *D. siamensis* venom displays weak pre-synaptic neurotoxicity and more myotoxicity in vitro. The neurotoxic and myotoxic effects of Chinese *D. siamensis* venom were largely neutralised by Chinese *D. siamensis* antivenom and Thai Russell’s viper antivenom. The non-specific *G. brevicaudus* and *D. acutus* antivenoms were only partially protective against the neurotoxic effects and ineffective against the myotoxic effects of Chinese *D. siamensis* venom. This suggests that the two non-specific antivenoms may not be appropriate for the treatment of envenomation by *D. siamensis*.

The t_50_ values and the inhibition of indirect twitches in vitro by Chinese *D. siamensis* suggest relatively weak neurotoxic effects compared to venom from Sri Lankan *D. russelii* [10] and ‘neurotoxic’ venoms more broadly [10,30,31,32]. Venoms that display the rapid inhibition of indirect twitches in skeletal muscle preparations (e.g., many elapid venoms; [30,31,32]) generally contain an abundance of short-chain α-neurotoxins. These rapidly acting toxins, which inhibit the binding of the neurotransmitter to the skeletal muscle nicotinic receptor, contribute to the very low t_50_/t_90_ values in comparison to venoms which lack α-neurotoxins. However, short-chain α-neurotoxins do not appear to play a major role in human envenomation [33].

Our results examining Chinese *D. siamensis* venom are in agreement with previous studies showing that the neurotoxic effects of Thai and Taiwanese *D. siamensis* venoms are relatively weak, mainly attributed to the presence of pre-synaptic neurotoxins [7,10,34]. The profiles of most *D. siamensis* venoms show little variation, with the majority of venom components being conserved across the geographical range [13,15,16,18,19,20,21,35] of the species. However, of interest is a pre-synaptic PLA_2_ toxin found in Sri Lankan *D. russelii* venom that appears to be a different subtype of toxin than those characterised in Eastern *D. siamensis* venom [17]. Although the Thai and Taiwanese species evoke similar neurotoxic effects in vitro, this is rarely clinically reported in envenomed humans. This is despite the fact that venoms possessing pre-synaptic neurotoxins most often cause neurotoxicity in humans. We have previously reported that U1-viperitoxin-Dr1a, the pre-synaptic neurotoxin from Sri Lankan Russell’s viper venom, constitutes approximately 19% of the whole venom [10]. However, while this toxin has a relatively high abundance, U1-viperitoxin-Dr1a has a relatively low potency in skeletal muscle preparations, compared to other pre-synaptic neurotoxins [30]. It is clear that the combination of quantity and potency of the pre-synaptic neurotoxins is important in the likely severity of paralysis in humans. In support of this hypothesis, it has been shown that neurotoxicity following envenomation by the Sri Lankan Russell’s viper was associated with high venom concentrations [11].

Some vipers (e.g., *D. russelii*, *Crotalus durissus terrificus*) can cause local or systemic myotoxicity, resulting in rhabdomyolysis. It is generally accepted that neurotoxicity and myotoxicity are unique manifestations following envenomation by *Daboia* species in Sri Lanka and South India. This is evidenced by several case studies in which almost half of the patients present with neurotoxicity [11]. Conversely, there appears to be lower rates of myotoxicity following envenomation by *D. siamensis* [21]. In the current study, Chinese Russell’s viper venom displayed concentration-dependent myotoxicity in the skeletal muscle preparation. These effects are likely to be attributable to the PLA_2_ component(s) of the venom. PLA_2_ activity in *Daboia* species venoms has previously been reported [7,10,12,14], and Tan et al. [16] confirmed the presence of putative PLA_2_ toxins in Guangxi *D. siamensis* venom. However, it is uncertain whether the PLA_2_ toxins of *D. siamensis* venom are responsible for in vitro myotoxic effects. This is due to the possibility of snake venom metalloprotease (SVMP)-mediated activity in systemic envenomation associated with *D. siamensis* venom, and the difficulty in correlating the role of specific toxins with human toxicity. Although *D. siamensis* venom has been shown to possess potent myotoxic activity in vitro, the relative frequency of myotoxicity and, hence, rhabdomyolysis in humans seems to be lower than that caused by Sri Lankan *D. russelii*. It has previously been shown that Sri Lankan *D. russelii* venom, and major PLA_2_ constituents (i.e., U1-viperitoxin-Dr1a and U1-viperitoxin-Dr1b), displayed weak in vivo or in vitro myotoxicity, respectively [9], while Thai *D. siamensis* [24] venom demonstrated more potent myotoxic effects than that of Sri Lankan venom. Interestingly, based on t_50_ values, Chinese venom is more potent than both Sri Lankan *D. russelii* and Thai *D. siamensis* venom. Prior in vitro studies have demonstrated that the venoms from black snakes, such as *Pseudechis australis* (Mulga snake), display potent myotoxic effects in the chick biventer [36], and the venom from Chinese *D. siamensis* was comparable to these effects at the same concentration. Given this, it suggests that *D. siamensis* envenomation could cause skeletal muscle damage, leading to more serious consequences, such as rhabdomyolysis [37]. Acute kidney injury caused by *D. siamensis* envenomation has frequently been linked to venom-induced consumption coagulopathy, haemolysis, and haemorrhage, and clinical signs have been attributable to subsequent glomerular obstruction combined with haemolysis and direct tissue destruction [38,39]. Unfortunately, there can be few conclusions about the myotoxic potency because there is insufficient information on the clinical effects in mainland China, since comprehensive clinical epidemiology data are lacking.

In mainland China, *D. siamensis* antivenom is not currently available for clinical use, and treatment entails the use of non-specific antivenoms. We investigated the efficacy of a new Chinese *D. siamensis* monovalent antivenom and compared this to the Russell’s viper antivenom from Thailand. We also examined the efficacy of the non-specific Chinese pit viper antivenoms against the neurotoxic and myotoxic effects of *D. siamensis* venom. These two Chinese pit viper antivenoms, raised against *Deinagkistrodon acutus* (sharp-nosed pit viper) and *Gloydius brevicaudus* (short-tailed pit viper), are used in the absence of *D. siamensis* antivenom, as recommended by the Chinese Expert Consensus in 2018. Although neuromuscular paralysis has notably been absent following *D. acutus* envenomation, and as flaccid paralysis may develop as a result of severe *G. brevicaudus* envenomation, the two antivenoms were only able to partially prevent the neurotoxic effects caused by Chinese *D. siamensis* venom. The weak efficacy of the antivenoms reflect a previous finding in which both pit viper antivenoms displayed weak immunoreactivity and cross-neutralising capabilities to Guangxi *D. siamensis* venom [16]. The fact that these antivenoms exert some protective effect indicates that similar components must be present in unrelated species for the antivenom to have efficacy. PLA_2_ toxins are likely to be responsible for the neurotoxic effects of *D. siamensis* venom, but the comparative venom proteomes of both *D. acutus* and *G. brevicaudus* show little similarity in the amino acid sequence of PLA_2_ subtypes when compared to *D. siamensis* venom [40,41]. It is therefore not surprising that there is only partial cross-reactivity, and thus weaker protective effects, of these antivenoms against *D. siamensis* venom-induced neurotoxicity.

Neither non-specific antivenom was effective at preventing myotoxicity induced by Chinese *D. siamensis* venom. While the venoms of these species are predominantly haemotoxic, the failure of the two Asiatic pit viper antivenoms to protect against the myotoxic effects of *D. siamensis* venom is not surprising given that pit viper venoms differ significantly to true viper venoms [42]. Pit viper venoms are also typically more damaging to tissue and muscles, with pathological symptoms not commonly seen following Chinese *D. siamensis* envenomation, including local ulceration [43]. This suggests that both *D. acutus* and *G. brevicaudus* antivenoms are not suitable for the treatment of *D. siamensis* envenomationand that specific *D. siamensis* antivenom is more appropriate in the absence of a Chinese *D. siamensis* antivenom. The prior addition of Thai *D. siamensis* monovalent antivenom prevented the neuromuscular blockade in the isolated skeletal muscle and myotoxicity.

Our study indicates that some of the antivenoms had a direct inhibitory effect on twitches. Therefore, all antivenoms were filtered to remove any potential contaminants. Preliminary work assessing both filtered and unfiltered antivenoms indicates that antivenom efficacy was not affected by this process. In addition, a larger concentration of antivenom (i.e., two to three times the recommended concentration) was required to neutralise the neurotoxic effects. Due to the high amounts of antivenoms used, filtration and concentration of the antivenoms enabled a reduction in the volume of antivenoms used in order to avoid altering the osmolarity of the organ bath solution.

Thai *D. siamensis* monovalent antivenom was almost equally as effective against the in vitro neurotoxic activity of Chinese *D. siamensis* venom. In contrast to the Guangxi *D. siamensis* venom used by Tan et al. [16], the *D. siamensis* venom used in our study originated from the Yunnan province, and the antivenom was developed against venoms of the same region, including Guangxi and Guangdong provinces [38,39] (Dr. Liang, personal communication with staff from the Shanghai Serum Biotechnology company). However, the presence of common antigens in the venom from geographical variants of *D. siamensis* is expected. Previous studies demonstrated substantial cross-reactivity between various *D. siamensis* antivenoms and geographical variants, implying shared epitopes in antigenic proteins [13,16,21,22].

We demonstrated that Chinese *D. siamensis* antivenom was able to prevent direct twitch reduction and the associated increase in baseline tension, albeit at four times the recommended antivenom concentration. Our preliminary studies demonstrated that lower concentrations of antivenom, of up to three times the recommended concentration, slightly attenuated the inhibition of direct twitches and partially prevented an increase in baseline tension. Additionally, Thai monovalent antivenom at twice the recommended concentration partially delayed the inhibition of direct twitches but completely prevented an increase in baseline tension. It was previously shown that Thai *D. siamensis* venom has primarily weaker myotoxic effects than Chinese venom at the same concentration in the chick biventer preparation [24], possibly due to a lower myotoxin content of Thai venom. However, the neutralising capabilities of the antivenom suggest that there are some antigenically similar toxins. While we have not confirmed the interaction between the Chinese and Thai antivenoms and the Chinese *D. siamensis* venom components (e.g., Western blot analysis) in this study, our in vitro neutralisation studies indicate that the antivenoms bind to key proteins involved in the neurotoxic and myotoxic effects of the venom.

As a result of common antigenic regions, the cross-neutralisation of snake venom toxins can occur with antivenoms that are raised against different species. This can occur with abundant toxins, such as PLA_2_ and SVMPs, as isoforms of different variations often share a high structural similarity at important or highly conserved regions [44]. PLA_2_ pre-synaptic neurotoxins and myotoxins are toxins that make up a major component of many snake venoms, including Russell’s viper, and can explain the venom activities of Chinese venom. Examples of biochemically characterised pre-synaptic PLA_2_ toxins include Daboiatoxin from Myanmar *D. siamensis* [12,14,15], Viperotoxin F from Taiwan D. *siamensis formensis* [7,12,19], and U1-viperotoxin-Dr1a and U1-viperotoxin-Dr1b from Sri Lankan *D. russelii* [10]. Pre-synaptic PLA_2_s from Thai *D. siamensis* venom have previously been identified as Russtoxin, but it is suggested they share a very similar pharmacological and structural profile to Viperotoxin F. It is logical to conclude that Chinese *D. siamensis* venom may be compositionally similar to Thai venom and *Daboia* species alike, suggesting that *D. siamensis* snakebite in Chinese regions may benefit from the clinical use of Chinese or possibly Thai *D. siamensis* antivenom.

## 4. Conclusions

We demonstrated that Chinese *D. siamensis* venom causes mild presynaptic neurotoxicity and myotoxicity, which is neutralized by both Chinese and Thai *D. siamensis* antivenom. However, the non-specific antivenoms against *D. acutus* and *G. brevicaudus* are markedly less effective. The efficacy of the Chinese *D. siamensis* antivenom supports ongoing development.

## 5. Materials and Methods

### 5.1. Animals

Male chicks (White Leghorn crossed with New Hampshire; Wagner’s Poultry, Coldstream, Victoria, Australia) were housed with free access to food and drinking water at Monash Animal Services (Monash University, Clayton, VIC, Australia).

### 5.2. Chemicals and Reagents

The following chemicals were purchased from Sigma-Aldrich, St. Louis, MO, USA: acetylcholine (ACh), carbamylcholine (CCh), d-tubocurarine chloride (dTC), and bovine serum albumin (BSA). The following chemicals were purchased from other companies: potassium chloride (KCl, Ajax Finechem Pty. Ltd., Taren Point, NSW, Australia). All chemicals were dissolved in MilliQ water unless stated otherwise.

### 5.3. Venoms and Antivenoms

Freeze-dried *Daboia russelii siamensis* venom was obtained from Orientoxin Co., Ltd. (LaiYang, Shandong, China). Venom was dissolved in 0.05% (*w*/*v*) bovine serum albumin (BSA) for experiments and stock was stored at −20 °C degrees until required. Chinese *D. siamensis* monovalent antivenom (batch number: 20200401; expiry date: 1 April 2022), Chinese *Deinagkistrodon acutus* antivenom (batch number: 10190101, expiry date: 18 June 2022), and Chinese *Gloydius brevicaudus* antivenom (batch number: 20190605, expiry date: 18/06/2022) were all purchased from Shanghai Serum Biological Technology Co., Ltd. (Shanghai, China). Thai *Daboia russelii siamensis* monovalent antivenom (batch numbers: WR00310, WR00119, WR00121; expiry dates: 7 July 2015, 3 September 2024, 19 February 2026) were purchased from Thai Red Cross Society, Bangkok, Thailand. Freeze-dried antivenom was reconstituted with the solution supplied by the manufacturer. The amount of each antivenom required to neutralise in vitro venom activities was based on the quantity of venom in the organ bath and the neutralising capability recommended by the manufacturer. According to manufacturer’s instructions: 1 µL of Chinese *D. russelii siamensis* antivenom neutralises 6 µg of *D. siamensis* venom, 136 U of *D. acutus* antivenom can neutralise 1–3 mg *of D. acutus* venom; 1500U of *G. brevicaudus* antivenom can neutralise 1–1.25 mg of *G. brevicaudus* venom; and 1 mL of Thai *D. russelii siamensis* antivenom can neutralise 0.6 mg of *D. siamensis* venom. All antivenoms were filtered prior to use. To start, 1 mL of antivenom was filtered using a 10 kDa centrifugal filter unit and centrifuged at 4000 rpm for 8 min. The supernatant was discarded and the retentate was stored at −20 °C until use. All antivenoms were concentrated; hence, the amounts required were adjusted (based on the neutralisation ratio and how much was required for a 5 mL organ bath) to be equivalent to the concentration of unfiltered antivenom.

### 5.4. Isolated Chick Biventer Cervicis Nerve–Muscle Preparation

Male chicks aged between 5 and 10 days old were euthanised following CO_2_ inhalation. Two tissues were dissected from each chick and mounted on wire tissue holders under a 1 g resting tension in 5 mL organ baths. Tissue preparations were maintained at 34 °C, and were bubbled with carbogen (95% O_2_ and 5% CO_2_) in a physiological salt solution containing 118.4 mM of NaCl, 4.7 mM of KCl, 1.2 mM of MgSO_4_, 1.2 mM of KH_2_PO_4_, 2.5 mM of CaCl_2_, 25 mM of NaHCO_3_, and 11.1 mM of glucose.

### 5.5. Neurotoxicity Experiments

Indirect twitches were evoked by stimulating the motor nerve (0.1 Hz; 0.2 ms) at supramaximal voltage (10–20 V) using an electrical stimulator. Twitches were recorded on a PowerLab system (AD Instruments Pty Ltd. Bella Vista, Australia) via a Grass FT03 force displacement transducer. Preparations were equilibrated for 20 min before the addition of dTC (10 µM). The abolishment of twitches confirmed selective nerve stimulation. To restore twitch responses, tissues were repeatedly washed with physiological salt solution. In the absence of electrical stimulation, contractile responses to exogenous ACh (1 mM for 30 s), CCh (20 µM for 60 s), and KCl (40 mM for 30 s) were then obtained. Following this, electrical stimulation was recommenced for 20–30 min until a steady twitch height was restored before venom or antivenom addition. Venom (10 and 30 µg/mL) was left in contact with the preparation for a maximum of 3 h. Where indicated, the ability of antivenom to prevent neurotoxicity was examined by the pre-addition of selected antivenoms for 20 min before venom addition. At the end of each experiment, the tissue was re-exposed to exogenous agonists ACh, CCh, and KCl, as previously indicated, to compare post-venom contractile responses against pre-venom responses.

### 5.6. Myotoxicity Experiments

For experiments examining the myotoxic effects of venom, the electrode was placed around the belly portion of the muscle of the preparation, and the preparation was directly stimulated (0.1 Hz; 2 ms) at a supramaximal voltage (20–30 V). Residual nerve-mediated twitches were abolished by the addition of dTC (10 µM) which remained in the organ bath throughout the experiment. After a stable twitch height was obtained, venom (10 or 30 µg/mL) was added to the preparation for a maximum period of 3 h. The efficacy of antivenoms was examined by the pre-addition of selected antivenoms for 20 min before venom addition.

### 5.7. Data Analysis and Statistics

For in vitro experiments, the twitch height was measured from baseline every 2 min after venom addition and expressed as a percentage of the pre-venom twitch height. In neurotoxicity studies, the time taken for 50% inhibition of twitches, expressed as the t_50_ value, was used to determine venom potency. Post-venom contractile responses to exogenous agonists ACh, CCh, and KCl, measured in grams, were expressed as a percentage of the initial pre-venom contractile responses. Data were normalised to a percentage, where the pre-venom agonist responses were taken as 100%, and the responses post-venom were measured as the percentage of their pre-venom response. For myotoxicity experiments, the changes in muscle baseline tension, measured in grams, were measured every 10 min after venom addition.

A one-way analysis of variance (ANOVA) was performed for comparisons between treatments. All ANOVA models were followed by Bonferroni’s multiple comparisons post-hoc test, where *p* < 0.05 was considered statistically significant. Comparisons of responses to exogenous agonists before and after the addition of venom or vehicle control were analysed using Student’s paired t-test. Data are presented as a mean ± standard error of mean (SEM) of n experiments. All data and statistical analyses were performed using PRISM 9.3.0 (GraphPad Software, San Diego, CA, USA, 2021).

## Data Availability

Data sharing not applicable.

## References

[1] Wüster W. (1998). The genus *Daboia* (Serpentes: Viperidae): Russell’s Viper. Hamadryad.

[2] Belt P.J., Malhotra A., Thorpe R.S., Warrell D.A., Wüster W. (1997). Russell’s viper in Indonesia: Snakebite and systematics. Symp. Zool. Soc. Lond..

[3] Ghose A., White J., Brent J., Burkhart K., Dargan P., Hatten B., Megarbane B., Palmer R. (2016). Asian Snakes. Critical Care Toxicology.

[4] Thorpe R.S., Pook C.E., Malhotra A. (2007). Phylogeography of the Russell’s viper (*Daboia russelii*) complex in relation to variation in the colour pattern and symptoms of envenoming. Herpetol. J..

[5] Alfred S., Bates D., White J., Mahmood M.A., Warrell D.A., Thwin K.T., Thein M.M., Sint San S.S., Myint Y.L., Swe H.K. (2019). Acute Kidney Injury Following Eastern Russell’s Viper (*Daboia siamensis*) Snakebite in Myanmar. Kidney Int. Rep..

[6] Hung D.Z., Wu M.L., Deng J.F., Lin-Shiau S.Y. (2002). Russell’s viper snakebite in Taiwan: Differences from other Asian countries. Toxicon.

[7] Wang Y., Lu P.J., Ho C., Tsai I. (1992). Characterisation and molecular cloning of neurotoxic phospholipases A_2_ from Taiwan viper (*Vipera russelli formosensis*). Eur. J. Biochem..

[8] Warrell D.A. (1989). Snake venoms in science and clinical medicine 1. Russell’s viper: Biology, venom and treatment of bites. Trans. R. Soc. Trop. Med. Hyg..

[9] Silva A., Johnston C., Kuruppu S., Kneisz D., Maduwage K., Kleifeld O., Smith A.I., Siribaddana S., Buckley N.A., Hodgson W.C. (2016). Clinical and Pharmacological Investigation of Myotoxicity in Sri Lankan Russell’s Viper (*Daboia russelii*) Envenoming. PLoS Negl. Trop. Dis..

[10] Silva A., Kuruppu S., Othman I., Goode R.J., Hodgson W.C., Isbister G.K. (2017). Neurotoxicity in Sri Lankan Russell’s Viper (*Daboia russelii*) Envenoming is Primarily due to U1-viperitoxin-Dr1a, a Pre-Synaptic Neurotoxin. Neurotox Res..

[11] Silva A., Maduwage K., Sedgwick M., Pilapitiya S., Weerawansa P., Dahanayaka N.J., Buckley N.A., Siribaddana S., Isbister G.K. (2016). Neurotoxicity in Russell’s viper (*Daboia russelii*) envenoming in Sri Lanka: A clinical and neurophysiological study. Clin. Toxicol..

[12] Gopalan G., Thwin M.M., Gopalakrishnakone P., Swaminathan K. (2007). Structural and pharmacological comparison of daboiatoxin from Daboia russelli siamensis with viperotoxin F and vipoxin from other vipers. Acta Crystallogr. Sect. D..

[13] Lingam T.M.C., Tan K.Y., Tan C.H. (2020). Proteomics and antivenom immunoprofiling of Russell’s viper (*Daboia siamensis*) venom from Thailand and Indonesia. J. Venom. Anim. Toxins Incl. Trop Dis..

[14] Thwin M.M., Gopalakrishnakone p., Yuen R., Tan C.H. (1995). A Major Lethal factor of the Venom of Burmese Russell’s Viper (*Daboia russelli siamensis*): Isolation, N-Terminal sequencing and biological activities of daboiatoxin. Toxicon.

[15] Risch M., Georgieva D., von Bergen M., Jehmlich N., Genov N., Arni R.K., Betzel C. (2009). Snake venomics of the Siamese Russell’s viper (*Daboia russelli siamensis*)—Relation to pharmacological activities. J. Proteome Res..

[16] Tan K.Y., Tan N.H., Tan C.H. (2018). Venom proteomics and antivenom neutralization for the Chinese Eastern Russell’s viper, *Daboia siamensis* from Guangxi and Taiwan. Sci. Rep..

[17] Tsai L.H., Lu P.J., Su J.C. (1996). Two types of Russell’s viper revealed by variation in Phospholipases A_2_ from venom of the subspecies. Toxicon.

[18] Yee K.T., Rojnuckarin P. (2020). Complementary DNA library of Myanmar Russell’s viper (*Daboia russelii siamensis*) venom gland. Comp. Biochem. Physiol. C Toxicol Pharmacol..

[19] Sanz L., Quesada-bernat S., Chen P.Y., Lee C.D., Chiang J.R., Calvete J.J. (2018). Translational Venomics: Third-Generation Antivenomics of Anti-Siamese Russell’s Viper, *Daboia siamensis*, Antivenom Manufactured in Taiwan CDC’s Vaccine Center. Trop. Med. Infect. Dis..

[20] Tan N.H., Fung S.Y., Tan K.Y., Yap M.K.K., Gnanathasan C.A., Tan C.H. (2015). Functional venomics of the Sri Lankan Russell’s viper (*Daboia russelii*) and its toxinological correlations. J. Proteom..

[21] Chaisakul J., Alsolaiss J., Charoenpitakchai M., Wiwatwarayos K., Sookprasert N., Harrison R.A., Chaiyabutr N., Chanhome L., Tan C.H., Casewell N.R. (2019). Evaluation of the geographical utility of Eastern Russell’s viper (*Daboia siamensis*) antivenom from Thailand and an assessment of its protective effects against venom-induced nephrotoxicity. PLoS Negl. Trop. Dis..

[22] Leong P.K., Tan C.H., Sim S.M., Fung S.Y., Sumana K., Sitprija V., Tan N.H. (2014). Cross neutralization of common Southeast Asian viperid venoms by a Thai polyvalent snake antivenom (Hemato Polyvalent Snake Antivenom). Acta Trop..

[23] Suntravat M., Yusuksawad M., Sereemaspun A., Perez J.C., Nuchprayon I. (2011). Effect of purified viper venom-factor X activator (RVV-X) on renal hemodynamics, renal functions, and coagulopathy in rats. Toxicon.

[24] Chaisukal J., Khow O., Wiwatwarayos K., Rusmili M.R.A., Prasert W., Othman I., Zainal Abidin S.A., Charoenpitakchai M., Hodgson W.C., Chanhome L. (2021). A Biochemical and Pharmacological Characterization of Phospholipase A_2_ and Metalloproteinase Fractions from Eastern Russell’s Viper (*Daboia siamensis*) Venom: Two Major Components Associated with Acute Kidney Injury. Toxins.

[25] Hung D.Z., Yu Y.J., Hsu C.L., Lin T.J. (2006). Antivenom treatment and renal dysfunction in Russell’s viper snakebite in Taiwan: A case series. Trans. R. Soc. Trop. Med. Hyg..

[26] Kalita B., Mackessy S.P., Mukherjee A.K. (2018). Proteomic analysis reveals geographic variation in venom compostion of Russell’s viper in the Indian subcontinent: Implications for clinical manifestations post-envenoming and antivenom treatment. Expert Rev. Proteom..

[27] Tan K.Y., Ng T.S., Bourges A., Ismail A.K., Khomvilai S., Sitprija V., Tan N.H., Tan C.H. (2020). Geographical variations in king cobra (*Ophiophagus hannah*) venom from Thailand, Malaysia, Indonesia and China: On venom lethality, antivenom immunoreactivity and in vivo neutralization. Acta Trop..

[28] Silva A., Hodgson W.C., Isbister G.K. (2016). Cross-Neutralisation of In Vitro neurotoxicity of Asian and Australian Snake Neurotoxins and Venoms by Different Antivenoms. Toxins.

[29] Tan C.H., Liew J.L., Ismail A.K., Maharani T., Khomvilai S., Sitprija V. (2017). Cross reactivity and lethality neutralization of venoms of Indonesia *Trimeresurus* complex species by Thai Green Pit Viper Antivenom. Toxicon.

[30] Barber C.M., Isbister G.K., Hodgson W.C. (2012). Solving the ‘Brown snake paradox’: In vitro characterisation of Australasian snake presynaptic neurotoxin activity. Toxicol. Lett..

[31] Liang Q., Huynh T.M., Ng Y.N., Isbister G.K., Hodgson W.C. (2021). In Vitro Neurotoxicity of Chinese Krait (*Bungarus multicinctus*) Venom and Neutralization by Antivenoms. Toxins.

[32] Wickramaratna J.C., Hodgson W.C. (2001). A pharmacological examination of venoms from three species of death adder (*Acanthophis antarcticus*, *Acanthophis praelongus* and *Acanthophis pyrrhus*). Toxicon.

[33] Silva A., Cristofori-Armstrong B., Rash L.D., Hodgson W.C., Isbister G.K. (2018). Defining the role of post-synaptic α-neurotoxins in paralysis due to snake envenoming in humans. Cell. Mol. Life Sci..

[34] Harvey A.L., Barfaraz A., Thomson E., Faiz A., Preston S., Harris J.B. (1994). Screening of snake venoms for neurotoxic and myotoxic effects using simple In-vitro preparations from rodents and chicks. Toxicon.

[35] Senji Laxme R.R., Khochare S., Attarde S., Suranse V., Iyer A., Casewell N.R., Whitaker R., Martin G., Sunagar K. (2021). Biogeographic venom variation in Russell’s viper (*Daboia russelii*) and the preclinical inefficacy of antivenom therapy in snakebite hotspots. PLoS Negl. Trop. Dis..

[36] Ramasamy S., Isbister G.K., Hodgson W.C. (2004). The efficacy of two antivenoms against the in vitro myotoxic effects of black snake (*Pseudechis*) venoms in the chick biventer cervicis nerve-muscle preparation. Toxicon.

[37] Ponraj D., Gopalakrishnakone P. (1997). Renal lesions in rhabdomyolysis caused by *Pseudachis australis* snake myotoxin. Kidney Int..

[38] (2018). Experts Group of Snake-bites Rescue and Treatment Consensus in China Expert Consensus on China Snake-bites rescue and Treatment. Chin. J. Emer. Med..

[39] Liang Q. (2021). Chinese Daboia siamensis Clinical Care and Symptoms.

[40] Gao J.F., Wang J., He Y., Qu Y.F., Lin L.H., Ma X.M., Ji X. (2014). Proteomic and biochemical analysis of short-tailed pit viper (*Gloydius brevicaudus*) venom: Age-related variation and composition-activity correlation. J. Proteom..

[41] Wang Y.M., Wang J.H., Tsai I.H. (1996). Molecular cloning and deduced primary structures of acidic and basic phospholipases A_2_ from the venom of *Deinagkistrodon acutus*. Toxicon.

[42] Tasoulis T., Isbister G.K. (2017). A review and Database of Snake Venom Proteomes. Toxins.

[43] Maduwage K., Hodgson W.C., Konstantakopolous N., O’Leary M.A., Gawarammana I., Isbister G.K. (2011). The in vitro toxicity of venoms from South Asian Hump-nosed pit vipers (Viperidae; *Hypnale*). J. Venom. Res..

[44] Chaisukal J., Rusmili M.R.A., Alsolaiss J., Albulescu L., Harrison R.A., Othman I., Casewell N.R. (2020). In Vitro Immunological Cross-Reactivity of Thai Polyvalent and Monovalent Antivenoms with Asian Viper Venoms. Toxins.

